# Erratum: Čapek et al. On the Weldability of Thick P355NL1 Pressure Vessel Steel Plates Using Laser Welding. *Materials* 2021, *14*, 131

**DOI:** 10.3390/ma14164598

**Published:** 2021-08-16

**Authors:** Jiří Čapek, Karel Trojan, Jan Kec, Ivo Černý, Nikolaj Ganev, Stanislav Němeček

**Affiliations:** 1 Department of Solid State Engineering, Faculty of Nuclear Sciences and Physical Engineering, Czech Technical University in Prague, Trojanova 13, 120 00 Prague, Czech Republic; karel.trojan@fjfi.cvut.cz (K.T.); nikolaj.ganev@fjfi.cvut.cz (N.G.); 2 Laboratory of Material Properties, SVÚM a.s., Tovární 2053, 250 88 Čelákovice, Czech Republic; kec@svum.cz (J.K.); ivo.cerny@seznam.cz (I.Č.); 3 Department of Laser Material Processing, RAPTECH s.r.o., U Vodárny 473, 330 08 Zruč-Senec, Czech Republic; nemecek@raptech.cz

The authors would like to correct the sentence in the “Materials and Methods” section of their article [1]: 

“The measurements were performed at CANAM infrastructure in Nuclear Physics Institute of the Czech Academy of Sciences (Řež, Czech Republic) using the SPN-100 Strain scanner.”

The authors would like to add the following sentence to the “Funding” section of their article [1]: 

“Neutron diffraction measurements were carried out at the CANAM infrastructure of the NPI ASCR Rez supported through MEYS project No. LM2015056. Presented neutron diffraction results were obtained with the use of infrastructure Reactors LVR-15 and LR-0, which is financially supported by the Ministry of Education, Youth and Sports—project LM2018120.”

The authors apologize to the Nuclear Physics Institute of the Czech Academy of Sciences and readers for any inconvenience caused. The authors state that the scientific conclusions are unaffected. The original article has been updated.
